# Threshold reduction and yield improvement of semiconductor nanowire lasers *via* processing-related end-facet optimization†

**DOI:** 10.1039/c9na00479c

**Published:** 2019-10-02

**Authors:** Juan Arturo Alanis, Qian Chen, Mykhaylo Lysevych, Tim Burgess, Li Li, Zhu Liu, Hark Hoe Tan, Chennupati Jagadish, Patrick Parkinson

**Affiliations:** Department of Physics and Astronomy and the Photon Science Institute, The University of Manchester Manchester UK patrick.parkinson@manchester.ac.uk; Department of Materials, The University of Manchester Manchester UK; Australian National Fabrication Facility, The Australian National University Canberra Australia; Department of Electronic Materials Engineering, Research School of Physics and Engineering, The Australian National University Canberra Australia

## Abstract

Both gain medium design and cavity geometry are known to be important for low threshold operation of semiconductor nanowire lasers. For many applications nanowire lasers need to be transferred from the growth substrate to a low-index substrate; however, the impact of the transfer process on optoelectronic performance has not been studied. Ultrasound, PDMS-assisted and mechanical rubbing are the most commonly used methods for nanowire transfer; each method may cause changes in the fracture point of the nanowire which can potentially affect both length and end-face mirror quality. Here we report on four common approaches for nanowire transfer. Our results show that brief ultrasound and PDMS-assisted transfer lead to optimized optoelectronic performance, as confirmed by ensemble median lasing threshold values of 98 and 104 μJ cm^−2^ respectively, with nanowires transferred by ultrasound giving a high lasing yield of 72%. The mean threshold difference between samples is shown to be statistically significant: while a significant difference in mean length from different transfer methods is seen, it is shown by SEM that end-facet quality is also affected and plays an important role on threshold gain for this nanowire architecture.

## Abstract 2

For nanowire lasers, end-facets matter; a rigorous statistical study demonstrates that short ultrasound or PDMA transfer provides optimized lasing performance.

## Graphical abstract

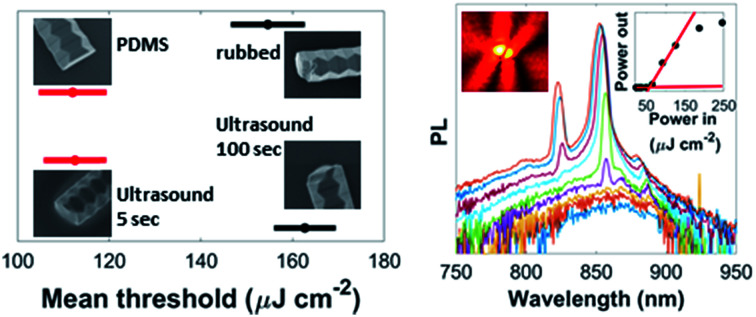

## 1 Introduction

Nanowires have been widely studied as coherent light sources for potential integration in the field of on-chip nanophotonics.^1–4^ Due to their characteristic elongated shape and reflective end facets, nanowires can act as a Fabry–Pérot resonant cavity under excitation.^5^ Recent progress has led to an increase in efficacy of semiconductor nanowire lasers through both improved nanowire geometry and optimized active region absorption.^6–14^ In many applications it is necessary to transfer the wires to a structured^15^ or lower index substrate such as silica aerogel,^16^ indium tin oxide (ITO)^7^ or SiO_2_,^17^ and, while many efforts have contributed to the improvement of lasing operation, the impact of different nanowire transfer techniques on nanowire geometry and, consequently optical performance has so far not been addressed.

Understanding the impact of processing steps on single nanowire lasers cannot be achieved through single or few wire measurements, as any systematic variation is masked by a high degree of wire-to-wire heterogeneity common to this architecture. Here we report on four of the most commonly used methods to transfer nanowires by investigating their effect on both geometry and optical performance using hundreds of single nanowire spectroscopic measurements for each method.^18,19^ Using automated data acquisition techniques combined with a robust statistical analysis is essential to identify correlations that would otherwise be impossible to resolve from a smaller set of measurements.^20^ Transfer methods used included a solution-based transfer by ultrasonication for 5 s and 100 s, a dry transfer by rubbing and PDMS stamping onto *z*-cut quartz substrates. The nanowires used for this study are a p-doped GaAs core-only architecture grown by Au-assisted vapor liquid solid mechanism as reported by Burgess *et al.*^8,19^ We compare the impact of transfer method on nanowire length, lasing yield (defined as the fraction of nanowires tested which display lasing behaviour) and lasing threshold, and correlate it with end-facet quality imaged using Scanning Electron Microscopy (SEM). We find that nanowires from sample transferred by 5 s ultrasound have the best lasing performance, with a median lasing threshold of 98 μJ cm^−2^ and a lasing yield of ∼72%, followed by PDMS with 104 μJ cm^−2^ and a reduced lasing yield of ∼60%. Additionally, we observe a statistically significant difference in mean nanowire length between the ultrasound and PDMS transferred samples of 2.68 ± 0.8 μm and 2.00 ± 0.65 μm respectively. While threshold gain is expected to have an inverse relationship with cavity length, this is not clearly observed here; the mean lasing threshold from nanowires transferred by PDMS is in fact comparable to 5 s ultrasonication, which we attribute to an improvement in end-facet quality in PDMS transfer as observed in SEM imaging. Nanowires transferred by rubbing or 100 s ultrasound suffer from lower optoelectronic performance due to poor end-facet quality or higher morphological degradation when exposed to a longer ultrasonication time.

## 2 Results and discussion

Four samples were prepared using nanowires originating from the same part of the same growth wafer: NW-US5, NW-US100, NW-R and NW-PDMS. Samples NW-US5 and NW-US100 were prepared by placing the growth substrate into iso-propylalcohol and dispersing nanowires into solution using a camSonix C575/T ultrasound bath (which uses a standard sine-wave modulation of 37 kHz and has a peak power of 600 W) for 5 and 100 seconds respectively, before drop depositing the solution onto clean quartz substrates. Sample NW-R was prepared by gently rubbing the as-grown nanowires directly onto quartz substrates, transferring wires using micro-mechanical cleavage. Sample NW-PDMS was prepared by using a cured PDMS stamp to peel nanowires from their growth substrate using gentle pressing. The nanowire-laden stamp was then pressed onto a quartz receiver substrate to adhere them. SEM images of free-standing nanowires on their growth substrate prior to being transferred are shown in the ESI. Since transferred nanowire densities were observed to vary between 200–1000 NWs mm^−2^, we randomly selected and studied 300–400 isolated nanowires from each sample to avoid overlapping wires.

**Fig. 1:**
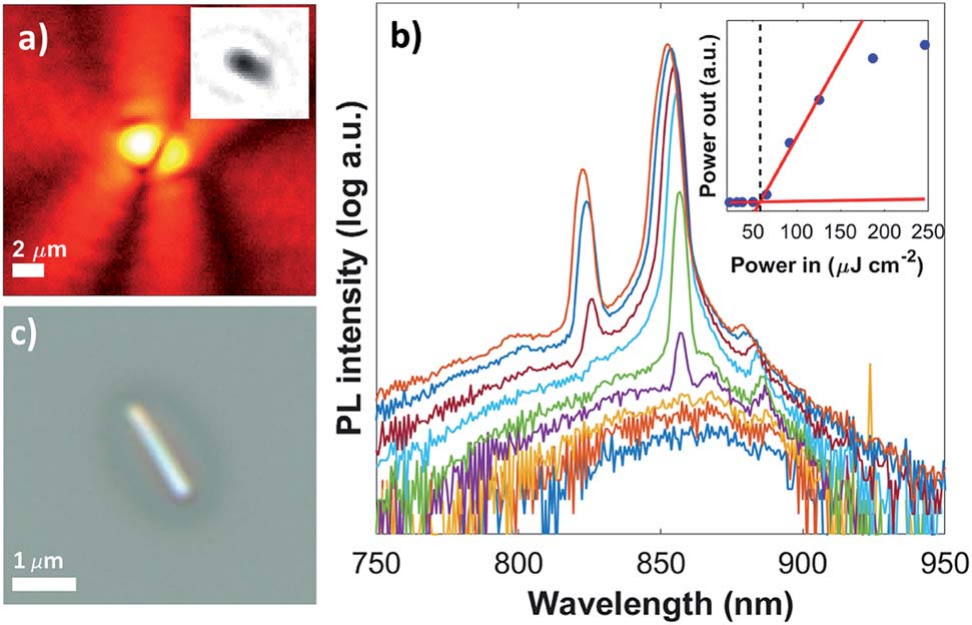
(a) Lasing emission image of a single nanowire from the US5 method under pulsed excitation; the inset shows the bright-field optical image used to estimate the length by machine vision on the same scale, and (b) corresponding power dependent emission spectra from the lasing emission. A light-in *versus* light-out plot is shown in the inset, where a linear fit is used for the spontaneous and stimulated emission regimes, and the calculated lasing threshold is indicated by a dashed line. (c) High-resolution optical image of a different nanowire prepared using the same method.

Nanowire length for each sample was measured by optical microscopy imaging; an example image of a randomly selected nanowire from sample NW-US5 is shown in the inset of Fig. 1a. Additionally, we studied lasing performance by power dependent photoluminescence at room temperature, using a 620 nm pulsed laser at a repetition rate of 250 kHz, and a 170 fs pulse duration. The beam was de-focused to a circular spot size of ∼360 μm^2^ to ensure uniform excitation along the whole nanowire. To avoid excitation of multiple nanowire lasers, we selected isolated nanowires with a minimum separation of at least 20 μm. Fig. 1a shows the emission image above lasing threshold (and the brightfield image, inset). The corresponding power dependent spectra from the same nanowire is shown in Fig. 1b. For comparison, a high-resolution optical image of another wire taken at higher magnification is shown in Fig. 1c. At low excitation energy a broad emission centred at ∼860 nm is observed. As the excitation fluence increases to ∼50 μJ cm^−2^ an initially narrow peak at 856 nm starts to emerge and continues to grow in intensity with excitation above threshold. The appearance of a secondary peak at 826 nm, associated with an additional longitudinal Fabry–Pérot cavity mode is observed at a fluence of ∼125 μJ cm^−2^. A blue shift in the main and second lasing peak is observed as a function of fluence, from 856 to 852 nm and 825 to 822 nm respectively. This blue shift is associated to the change in material refractive index induced by the increased photo-excited carrier density, as previously reported for GaAs.^6,8,21–23^ At high excitation fluence, we observe a lasing peak line-width of ∼10 nm, which is larger than that seen just above threshold (and larger than typically observed for nanowire lasers). We attribute this large line-width to a smearing effect caused by the carrier density (and hence effective refractive index) varying during the laser emission.^24^ The light-in *vs.* light-out (LILO) plot is shown in the inset of Fig. 1, where a linear fit was used for the spontaneous emission and stimulated emission regimes, and the lasing threshold was taken as the intersection of the two lines as previously described^18^ (58 μJ cm^−2^ for this wire).

**Fig. 2:**
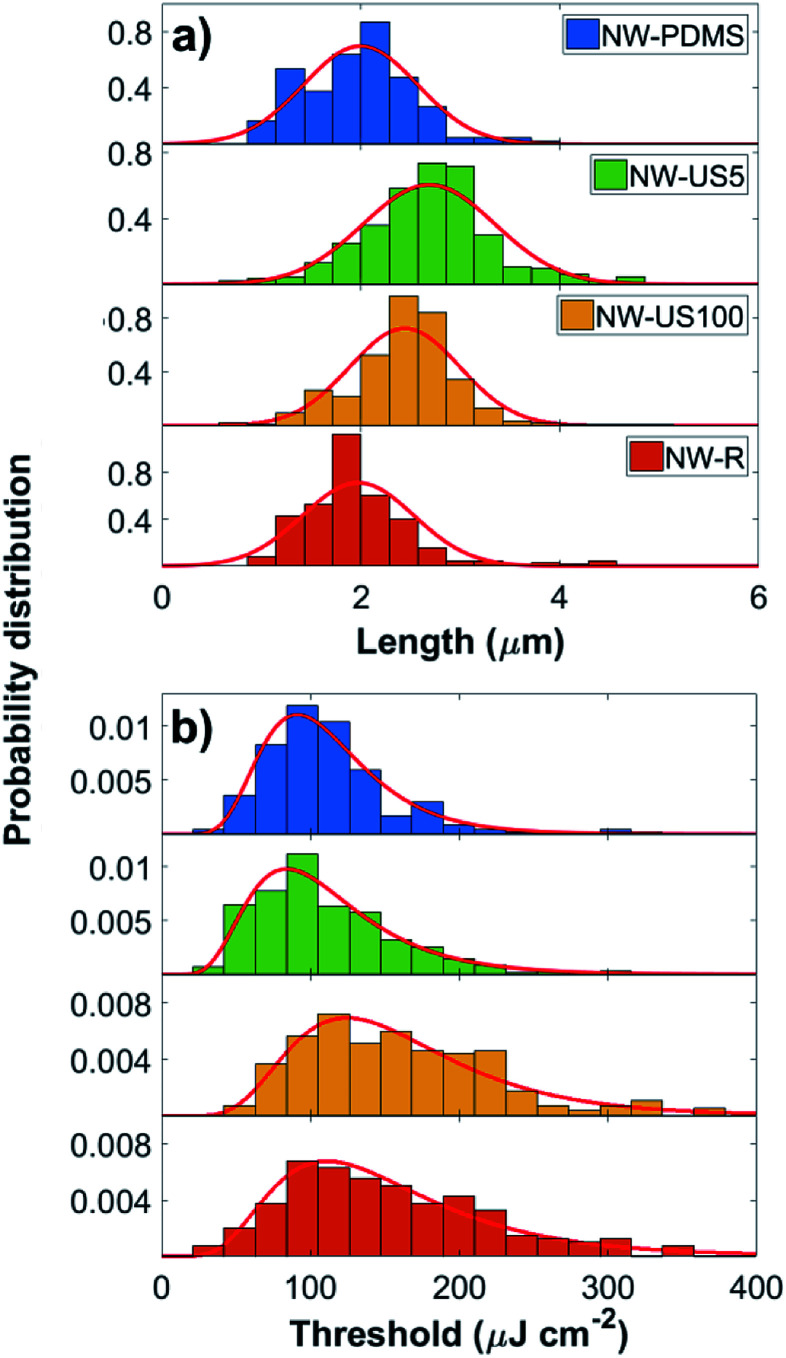
(a) Length distribution measured from optical imaging and (b) lasing threshold distributions corresponding to each sample. The red line indicates the normal distribution function fit for the length data, and a log-normal distribution fit to the threshold data. A summary of the parameters for the fits in (a) and (b) can be found in Table 1.

Fig. 2a shows the length histograms corresponding to each sample, where a normal probability distribution function is used to fit the data. Samples NW-US5 and NW-US100 had the longest average length with values of 2.68 ± 0.8 μm and 2.44 ± 0.65 μm respectively, whereas samples NW-PDMS and NW-R have a mean length of 2.00 ± 0.65 μm and 1.97 ± 0.65 μm respectively. This result indicates that nanowires transferred by solution-based methods seem to fracture closer to the base resulting in longer wires. These values were confirmed by SEM, where we measured 12 and 8 randomly selected nanowires from samples transferred by PDMS and rubbing respectively, and 6 nanowires transferred by ultrasound for both 5 s and 100 s (details in the ESI†).

Fig. 2b shows lasing threshold histograms corresponding to each sample where the log-normal probability density function (PDF) is represented by a red line; median values are summarized in Table 1. We found sample NW-US5 to have the lowest median lasing threshold (*T*_m_) with a value of 98^+40^_−23_ μJ cm^−2^ and a lasing yield of 72%, where the lasing yield is taken as the ratio between nanowires showing lasing (without undergoing thermal degradation), divided by the total number of wires studied for that sample. Sample NW-PDMS also had a low median threshold of 104^+27^_−21_ μJ cm^−2^; however it had a lower lasing yield of 60%. The lower yield could come from the nature of PDMS stamping where a number of shorter and heavily tapered nanowires are picked up during the transfer process. These pseudo-nanowire structures were observed in SEM imaging on two separate PDMS samples but not found when using any other transfer methods (details in the ESI†). While samples NW-R and NW-US100 also have high lasing yields (72% and 76%) they showed an increase in median threshold with values of 139^+60^_−38_ and 157^+48^_−46_ μJ cm^−2^ respectively and the largest spread in measured threshold values (Table 1). We note that the mean threshold from sample NW-US5 is higher than the one reported by us in previous works for the same growth.^19^ Given that the samples used for this study were prepared from a different part of the same growth substrate, we attribute this difference to variation in nanowire growth as a function of position.

**Table 1:** Summary of lasing yield, threshold (with error bars indicating interquartile range) and mean lengths for each studied transfer technique

Method	Yield (%)	*T*_m_ (μJ cm^−2^)	Champion (μJ cm^−2^)	Mean length (μm)
NW-US5	72	98^+40^_−23_	38	2.68 ± 0.8
NW-US100	76	157^+48^_−46_	49	2.44 ± 0.65
NW-R	72	139^+60^_−38_	31	1.97 ± 0.65
NW-PDMS	60	104^+27^_−21_	28	2.00 ± 0.65

To verify that the length and threshold difference across all samples is statistically significant we performed an analysis of variance (ANOVA) and Tukey–Kramer tests,^25^ where each sample mean is denoted as *μ*_PDMS_, *μ*_R_, *μ*_US5_ and *μ*_US100_. ANOVA is used to compare two or more means from independent (unrelated) groups using an F-distribution. The null hypothesis *H*_0_ for the test is that all the means are equal, in this case

1 *H*_0_: *μ*_PDMS_ = *μ*_R_ = *μ*_US5_ = *μ*_US100_

**Fig. 3:**
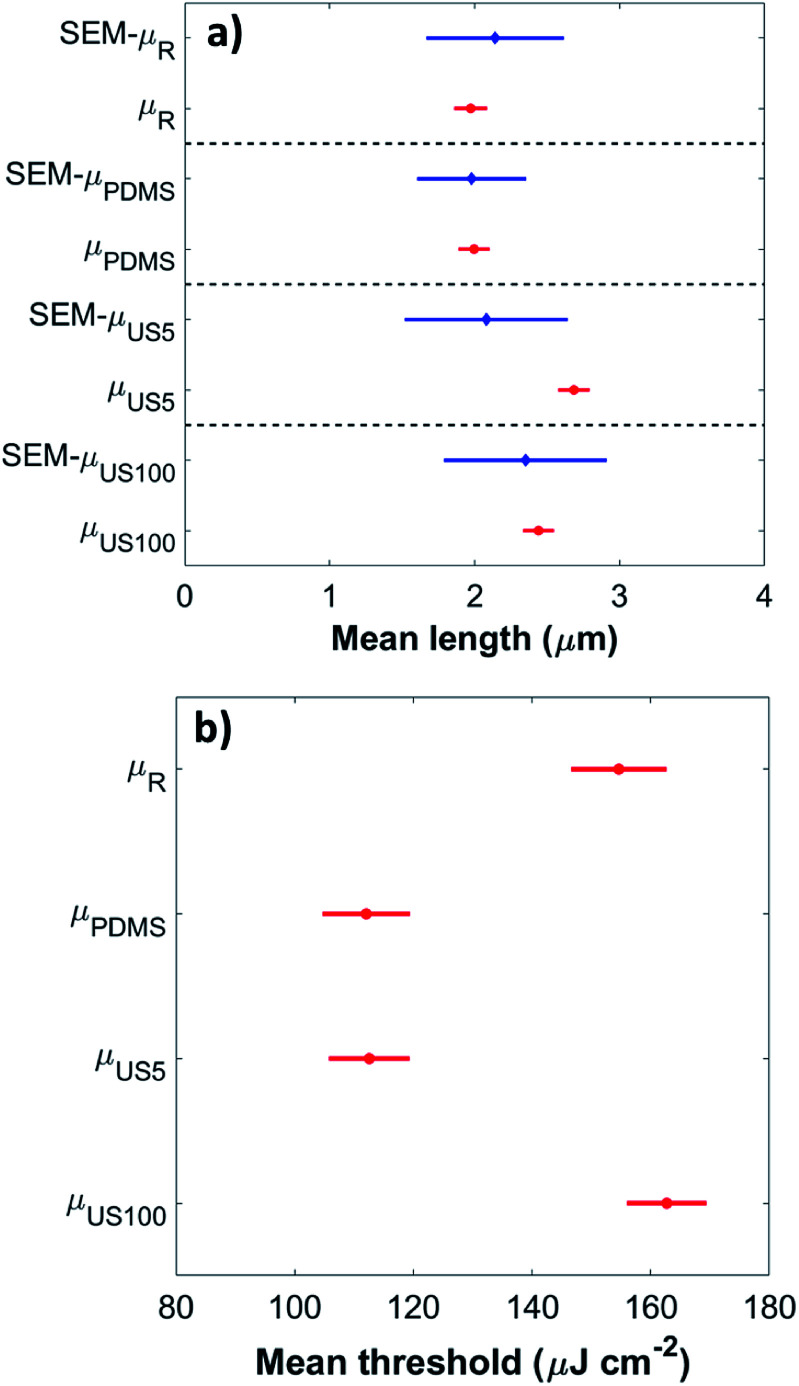
(a) Mean length calculated from ANOVA and T–K tests, red points correspond to mean length from optical microscopy and blue diamonds correspond to length measured by SEM imaging. (b) Lasing threshold mean values calculated from ANOVA and T–K tests. In both (a) and (b) the bars indicate the standard error corresponding to each group.

The alternative hypothesis *H*_1_ would state that the means are not equal. Therefore, if any of the means are statistically unequal *H*_0_ is said to be rejected and the test provides a significant result. For our study we ran two ANOVA tests: one on the mean length and another on the mean threshold across all groups. In both cases *H*_0_ was rejected with a statistically significant evidence at *p* ≪ 0.01 which shows a difference in mean length and lasing threshold among the four transfer methods (further details of the one-way ANOVA calculations are given in the ESI†). To identify which means are statistically different, a Tukey–Kramer (T–K) ad-hoc test was used. Fig. 3a shows the graph of mean length calculated from ANOVA and T–K tests. It can be seen that there is a significant difference in mean length obtained by optical microscopy between samples transferred by ultrasonication (*μ*_US5_ and *μ*_US100_) and samples transferred mechanically (*μ*_PDMS_ and *μ*_R_). Fig. 3b shows the graph of ANOVA and T–K tests for mean threshold, where sample means *μ*_PDMS_ and *μ*_US5_ show a statistically significant reduction in threshold values when compared with the other methods.

**Fig. 4:**
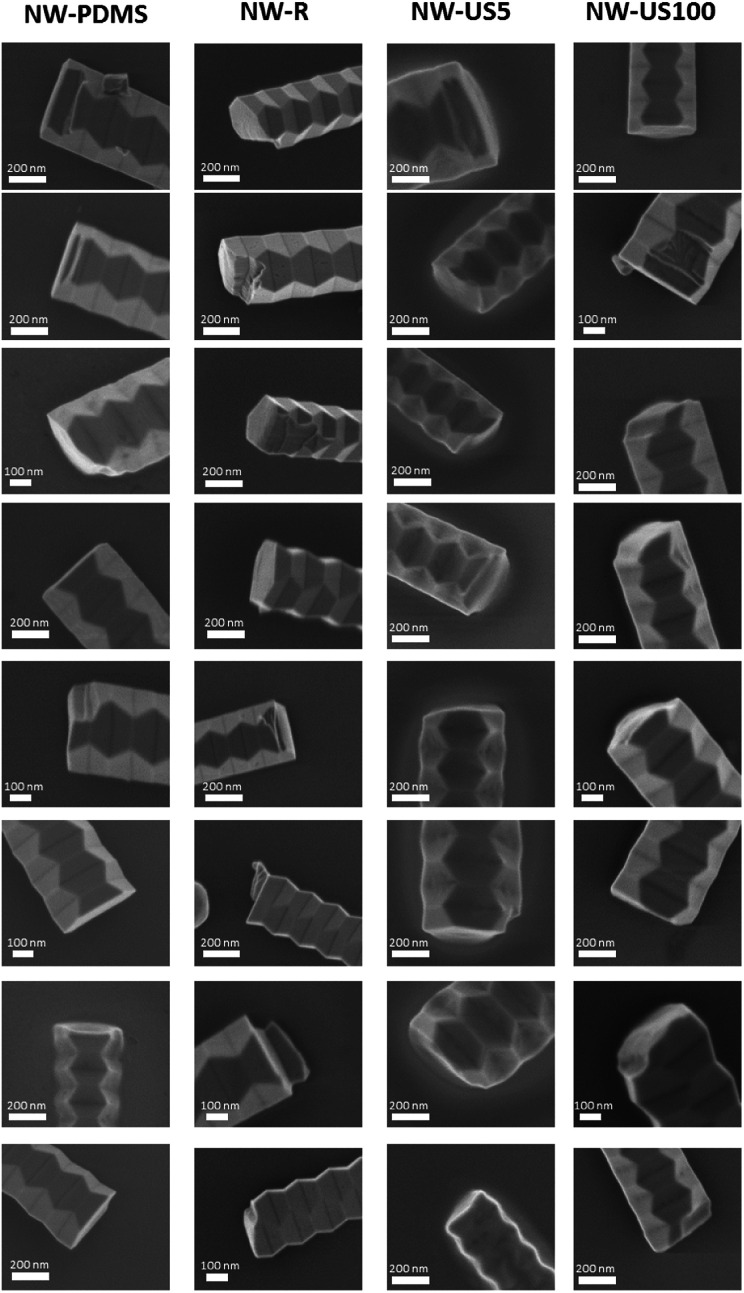
SEM imagery of the bases of a number of nanowires. Nanowires from sample NW-PDMS show a predominantly smooth and flat fracture point at the base.

The variation in performance across all samples can be understood from the difference in threshold gain (*g*_th_). By approximating a nanowire to a cylindrical Fabry–Pérot cavity,^26^*g*_th_ can be expressed as2
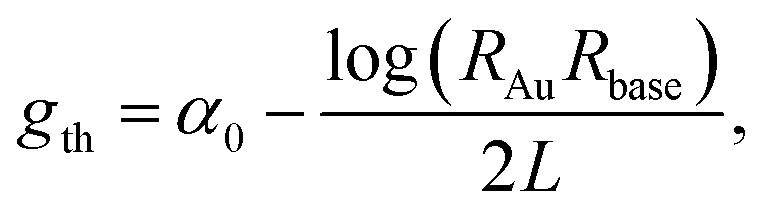
where *α*_0_ is the distributive losses *R*_Au,base_ the reflectivity coefficients at the Au tip and base of the nanowire respectively and *L* is the cavity length. The low mean lasing threshold from sample NW-US5 is attributed to the increased cavity length whereas sample NW-PDMS, while having length values ∼25% lower but comparable mean threshold, could stem from an improvement in the quality of the nanowire end-facet. Since the Au nanoparticle present in these wires is expected to improve the reflectivity,^6^ and assuming *R*_Au_ to be constant across all wires, it is likely that the decrease in threshold from sample NW-PDMS comes from an improvement in facet quality, and therefore *R*_base_, at the base of the nanowire. To verify end-facet structure we prepared four samples by transferring nanowires to Si wafers using the methods described above and imaged the base of randomly selected nanowires from each sample by SEM. Fig. 4 shows a difference in end facet quality corresponding to each transfer method. A close inspection of nanowires from samples NW-PDMS show a predominantly flat and smooth surface compared to sample NW-R, which could be contributing to the reduction in threshold gain. An alternative approach to visualise the end facet quality associated with each transfer method is provided by examining the exfoliated growth substrates using SEM after nanowire transfer (data and details in the ESI†). After transfer, breaking points resulting from PDMS stamping show predominantly smoother facets when compared with substrates after ultrasound or mechanical rubbing, further supporting the higher cavity quality achievable using the PDMS method. Despite sample NW-US100 having longer lengths, the higher mean threshold suggests that nanowires exposed to longer ultrasonication may be subject to heavier damage. Indeed, crystal dislocation and twinning propagation has been reported for GaAs nanowires under high tensile strain.^27^

## 3 Conclusion

In conclusion, we report optoelectronic performance of p-doped GaAs nanowire lasers transferred by four commonly used methods: mechanical rubbing, PDMS stamping and ultrasound for 5 and 100 s. By comparing the mean lasing threshold from each sample using ANOVA, we found nanowires transferred by a 5 s ultrasound bath and PDMS to have a statistically-significant lower threshold and a lasing yield of ∼72% and 60% respectively. While a significant variation in length was observed between these two samples, we attribute threshold gain reduction from samples transferred by PDMS to the improvement in end-facet quality at the base of nanowires as confirmed by SEM. For this reason, we suggest that PDMS stamp-transfer is the preferred method when single nanowire laser devices are required, and particularly where nanowire selection is possible.^28^ We note that while this study reflects the effect of different transfer methods on nanowire performance for a specific architecture, the large scale optical technique here used can be extended to a wide range of applications for nanowire characterization. We thus demonstrate a new robust methodology for understanding fabrication processing for bottom up nanotechnology towards high yield nanolasing.

## Conflicts of interest

There are no conflicts to declare.

## Supplementary Material

NA-001-C9NA00479C-s001
