# Supplementary material for: Threshold reduction and yield improvement of semiconductor nanowire lasers via processing-related end-facet optimization
Source: Nanoscale Adv. 2019 Oct 2;1(11):4393–7. doi: 10.1039/c9na00479c (PMC9417496; doi:10.1039/c9na00479c)
Supplement: NA-001-C9NA00479C-s001 [file NA-001-C9NA00479C-s001.pdf]

Electronic Supplementary Material (ESI) for Nanoscale Advances.
This journal is © The Royal Society of Chemistry 2019

Supporting information for: Threshold reduction and yield improvement of semiconductor nanowire lasers via processing-related end-facet optimization

Juan Arturo Alanis,[†] Qian Chen,[‡] Mykhaylo Lysevych,[¶,§] Tim Burgess,[§] Li Li,[¶,§] Zhu Liu,[‡] Hark Hoe Tan,[§] Chennupati Jagadish,[§] and Patrick Parkinson[*,†]

†*Department of Physics and Astronomy and the Photon Science Institute, The University of Manchester, Manchester, UK*

‡*Department of Materials, The University of Manchester, Manchester, UK*

¶*Australian National Fabrication Facility, The Australian National University, Canberra, Australia*

§*Department of Electronic Materials Engineering, Research School of Physics and Engineering, The Australian National University, Canberra, Australia*

E-mail: patrick.parkinson@manchester.ac.uk

Scanning Electron Microscopy

Fracture point imaging

Figure S1 shows Scanning Electron Microscope (SEM) images of growth substrates before and after each transfer process respectively.The top row show nanowires on their original growth substrate prior to being transferred onto quartz. Note that both true nanowires (with a superlattice structure) and pseudo-nanowires (with no gold seed) can be observed on the substrate. All subsequent rows include images from growth substrates showing nanowire fracture point associated to each transfer method (as labelled). Panels on the left show high resolution images associated to each label.

Pseudo-nanowire structures on SEM-PDMS

The lower lasing yield of 60 % from sample NW-PDMS is attributed to the presence of pseudo-nanowire objects which could derive from the nature of PDMS stamping for this nanowire architecture. These objects were observed to be present on nanowires transferred by PDMS when performing SEM for length calculations; a few examples are shown in Figure S2. The geometry of these structure is clearly not optimized for lasing; moreover, the lack of a clear gold colloid at the tip indicates that these are seed-free catalyzed wires; possibly catalyzed by defects during the growth. It is not clear why these wires are not transferred by other techniques, however, their short length and thick base are likely to make them more mechanically stable than the true nanowires.

Length calculation from Scanning Electron Microscopy

An additional length study was performed by SEM to compare with the large scale optical study outlined in the main text. For the SEM study 4 samples were prepared by transferring nanowires onto a Si substrate using the transfer methods detailed in the main text and labeled SEM-PDMS, SEM-R, SEM-US5 and SEM-US100. Figure S3 shows a few sample

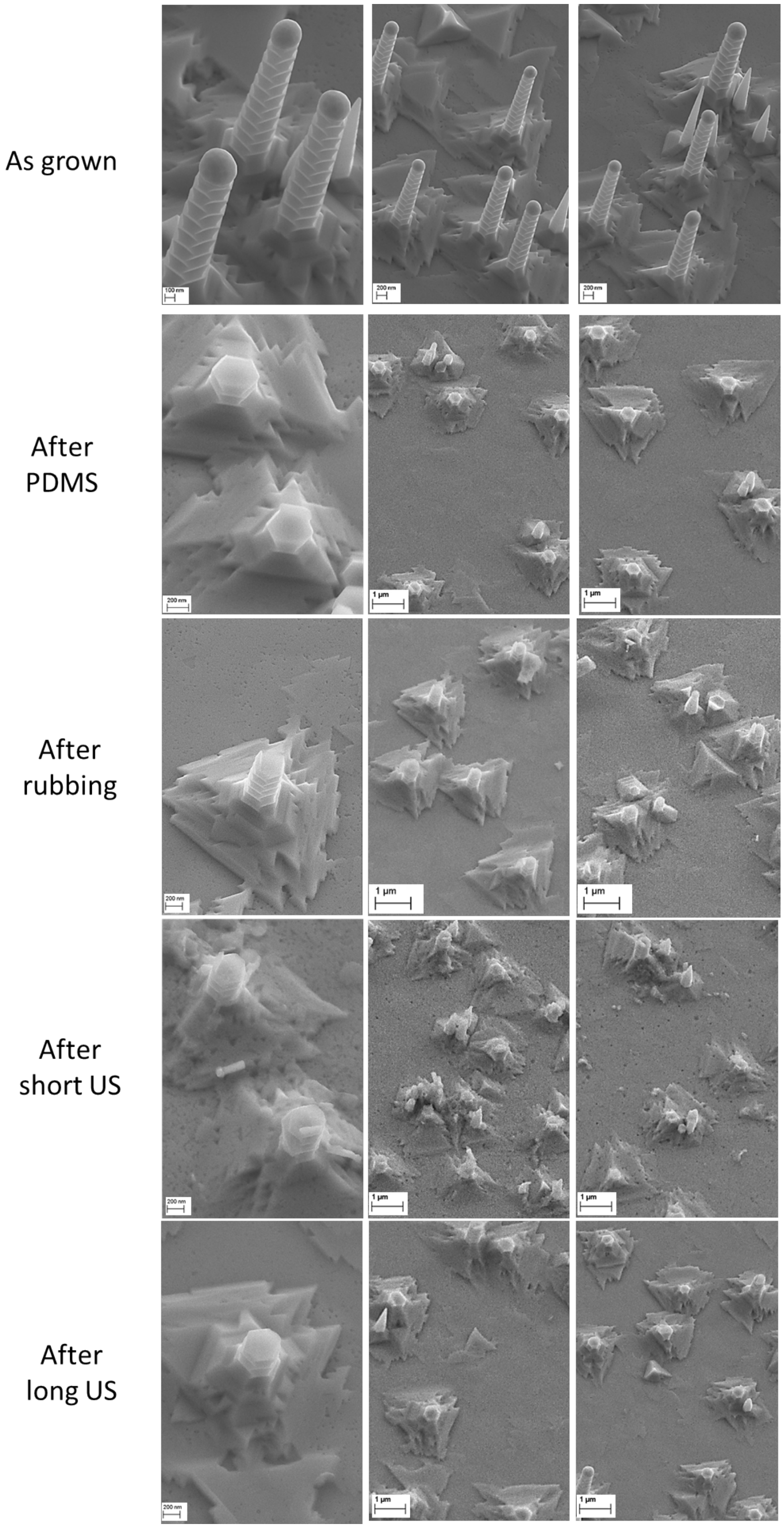

Figure S1: SEM imagery of free standing nanowires on their original growth substrate (Top row), and fracture point after nanowire transfer corresponding to each transfer method (left, top to bottom). (Center and Right columns) lower magnification images corresponding to each row.

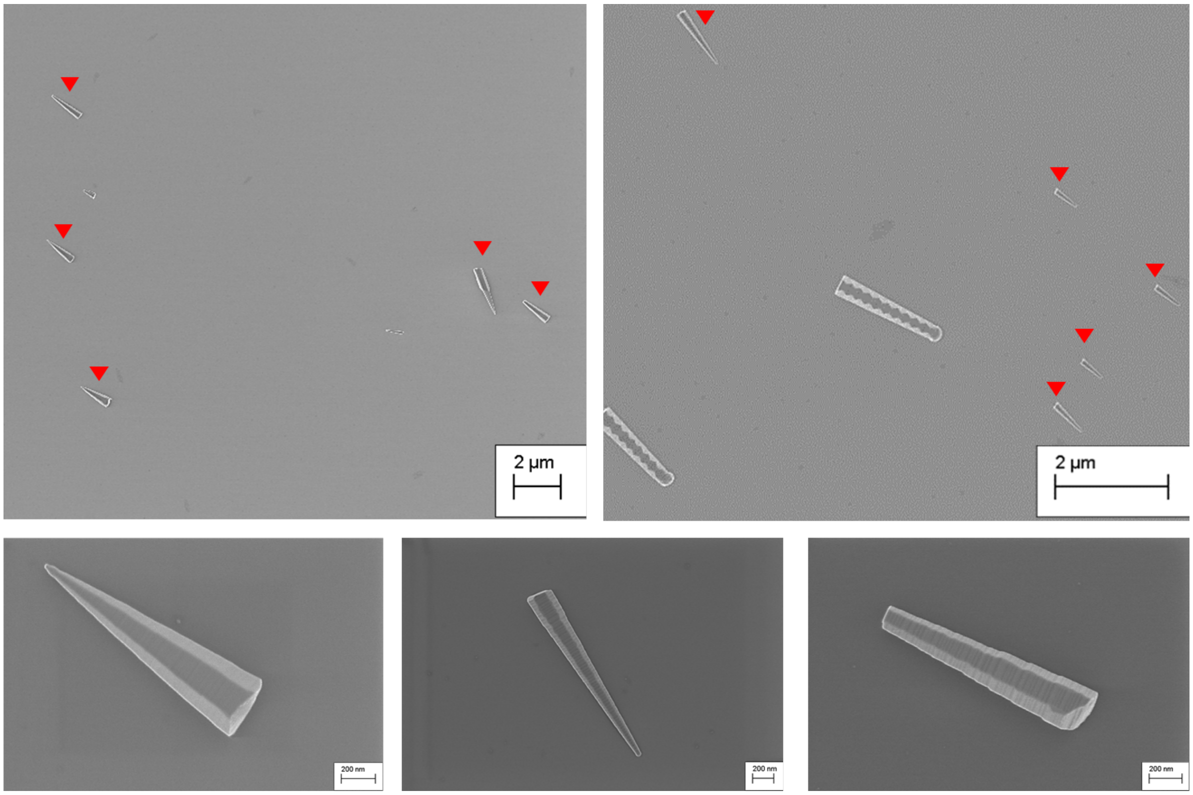

Figure S2: SEM imagery of a few non-wire structures found on sample SEM-PDMS. The two top insets show images at low magnification, where such nanostructures are flagged with red triangles. The three bottom insets show magnified images from three randomly selected nanostructures. We attribute the lower lasing yield of 60 % from sample NW-PDMS to the presence of these pseudo-nanowire objects.

images from nanowires randomly selected corresponding to each sample. A summary of lengths and mean values is shown in Table S1.

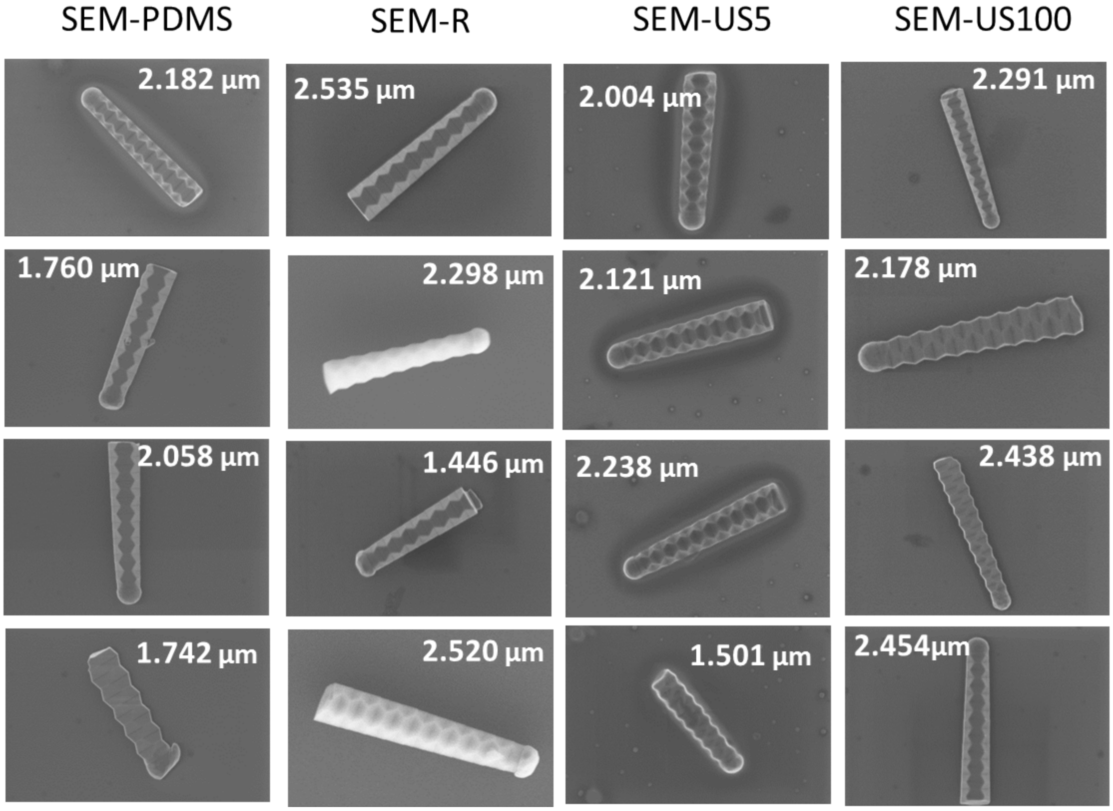

Figure S3: SEM imagery of a few selected nanowires taken from each sample. The measured length corresponding to each wire is displayed.

ANOVA

Calculations

For the ANOVA study we considered four independent groups corresponding to each transfer method: NW-R, NW-PDMS, NW-US5 and NW-US100. We wish to test whether there is a statistically significant difference in mean length and mean lasing threshold among the four groups. The hypotheses to be tested in this study are

Table S1: SEM length summary.

	SEM-PDMS	SEM-R	SEM-US5	SEM-US100
	2.154	1.958	1.501	2.407
	2.027	2.298	2.321	2.454
	2.034	2.520	2.238	2.438
	1.808	2.218	2.121	2.178
	1.742	2.224	2.004	2.291
	1.344	1.931	2.297	2.327
	2.058	1.446		
	1.760	2.535		
	2.128			
	2.182			
	2.341			
	2.173			
**Mean**	1.97±0.17	2.14±0.21	2.08±0.24	2.34±0.24

$$H_0 : \mu_{PDMS} = \mu_R = \mu_{US5} = \mu_{US100} \tag{1}$$

and

$$H_1 : \text{At least one mean is different.} \tag{2}$$

The test statistic F for testing H_0: μ_1=μ_2=...=μ_k is given by the ratio

$$F = \frac{\text{MSB}}{\text{MSW}} \tag{3}$$

where MSB is the mean squares between groups obtained from the sum of squares (SSB) and degrees of freedom (DoF_1) such that

$$\text{MSB} = \frac{\text{SSB}}{\text{DoF}_1} = \frac{\sum\limits_{j=1}^{k} n_j(\mu_j - \mu_{tot})^2}{k-1} \tag{4}$$

where n_j is the sample size in the jth group, μ_j is the sample mean, μ_{tot} is the total mean and k is the number of independent groups. MSW is the mean squares within groups obtained from its sum of squares (SSW) and degrees of freedom (DoF_2) written as

$$\text{MSW} = \frac{\text{SSW}}{\text{DoF}_2} = \frac{\sum_{j=1}^{k}\sum_{j=1}^{k}(X-\mu_j)^2}{N-k} \tag{5}$$

where X is an individual observation and N is the total number of observations in the study. The critical value to reject H_0 is found in a table of probability values for the F distribution which is found in literature.

Mean length ANOVA results

An ANOVA was used to compare mean length measured by optical imaging. The sample means corresponding to each transfer method are denoted μ_{PDMS}, μ_R, μ_{US5} and μ_{US100} as shown in the main manuscript. Nanowire mean length from samples measured by SEM were also included in the same study (denoted SEM-μ_{PDMS}, SEM-μ_R, SEM-μ_{US5} and SEM-μ_{US100} in the main manuscript). Table S2 summarizes ANOVA results where H_0 is rejected at a significance level $p \ll 0.01$.

Table S2: Mean length ANOVA results

**Source of Variance**	**SS**	**DoF**	**MS**	F	p
Between groups	105.412	7	15.0589	44.33	6.61×10^{-56}
Within groups	404.878	1192	0.3397		
Total	510.29	1199			

Mean threshold ANOVA results

An ANOVA test was used to verify if the difference in mean threshold was statistically significant across all transfer methods. The sample means were denoted μ_{PDMS}, μ_R, μ_{US5} and μ_{US100} as shown in the main manuscript. Table S3 shows the summary for ANOVA calculations where H_0 was rejected at significance $p \ll 0.01$.

Table S3: Mean threshold ANOVA results

**Source of Variance**	**SS**	**DoF**	**MS**	**F**	**p>F**
Between groups	530526.1	3	176842	48.34	4.62×10^{-29}
Within groups	3450015.6	943	3658.6		
Total	3980541.7	946			
